# 886. Impact of Race on the Early Diagnosis and Treatment of Lyme Disease

**DOI:** 10.1093/ofid/ofad500.931

**Published:** 2023-11-27

**Authors:** Samuel J Starke, Alison Rebman, John Miller, John Aucott

**Affiliations:** Johns Hopkins University School of Medicine, Baltimore, Maryland; Johns Hopkins University School of Medicine, Baltimore, Maryland; Johns Hopkins University School of Medicine, Baltimore, Maryland; Johns Hopkins University School of Medicine, Baltimore, Maryland

## Abstract

**Background:**

Previous studies using single-state and Medicare surveillance data found that Black patients with Lyme Disease (LD) are more likely to present outside of the typical summer months and have higher rates of disseminated disease at diagnosis. We further explored differences in clinical presentation and early treatment of Lyme Disease by self-reported race among a large cohort of patients at an academic research clinic.

**Methods:**

We abstracted data from all patients with untreated or post-treatment LD seen at the Johns Hopkins Lyme Disease Research Center (LDRC)—either for clinical care or research participation. Patients self-reported their demographics, and clinical information regarding their initial Lyme presentation was either recorded at the time of diagnosis or was abstracted from prior, external medical records. We examined the distribution of race across initial LD clinical presentations—categorized as 1) erythema migrans (EM) rash only, 2) disseminated disease, or 3) non-specific symptoms only—using chi-squared or Fisher’s exact test and fit logistic regression models. We compared racial differences in time to appropriate treatment using a one-sided Mann-Whitney test.

**Results:**

Of 1363 individuals seen at the LDRC, 93% were white, 3% were Black, 2% were Asian, and 3% self-identified as either ‘other’ or more than one race. Compared to white patients, Black patients were less likely to present with erythema migrans (EM) rash (p < 0.0001) and more likely to present with disseminated disease or non-specific symptoms (*p*=0.009, *p*=0.019, respectively). Black patients were more likely to have received a course of inappropriate antibiotic therapy ( 16.7% vs 8.0 %, *p*= 0.058). Also, Black patients with EM rash experienced longer time to appropriate treatment (16.3 days vs 4.0, *p*=0.062).

**Figure:**
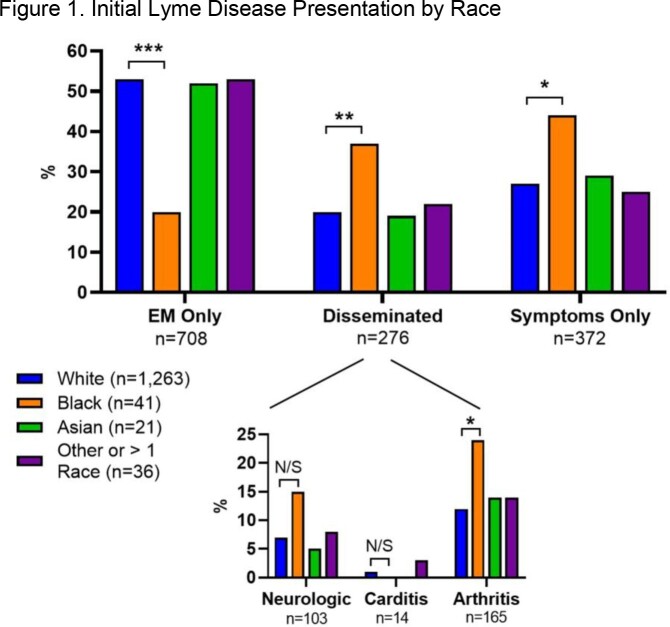

Number of asterisks indicates degree of statistical significance. N/S means "non-significant"

**Figure:**
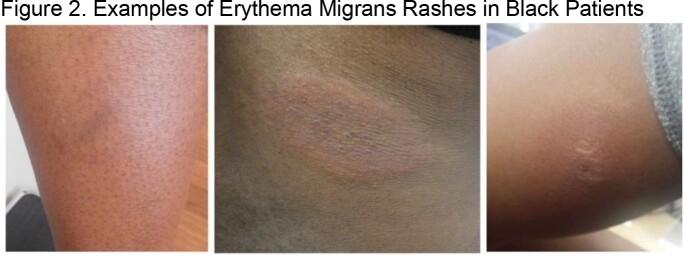

**Conclusion:**

We identified several racial disparities in the early detection and treatment of Lyme Disease, highlighting the need for increased awareness of Lyme and its early manifestations among patients of color and the front-line clinicians who care for them. Because darker skin tones are generally under-represented in medical textbooks and patient education materials, we include several examples of EM rashes from Black patients seen at the LDRC (Figure 2).

**Disclosures:**

**John Aucott, MD**, Pfizer, Inc.: Advisor/Consultant

